# 5,11,17,23-Tetra­kis(1,1-di­methyl­eth­yl)-26,28-di­hydroxy­calix[4]arene-25,27-mono­thia­crown-3

**DOI:** 10.1107/S1600536813008684

**Published:** 2013-04-10

**Authors:** De-Xun Xie, De-Lie An

**Affiliations:** a Department of Chemistry, College of Chemistry and Chemical Engineering, Hunan University, Changsha 410082, People’s Republic of China

## Abstract

The title calix[4]arene compound [systematic name: 3,9,15,34-tetra-*tert*-butyl-19,29-dioxa-24-thia­hexa­cyclo­[15.13.7.1^7,11^.1^32,36^.0^5,30^.0^13,18^]nona­triaconta-1(30),2,4,7,9,11(39),13,15,17,32,34,36(38)-dodeca­ene-38,39-diol], C_52_H_70_O_4_S, displays a cone-like conformation, the opposite arene rings bridged by the mono­thia­crown-3 unit are nearly parallel [dihedral angle = 16.01 (18)°], whereas the other opposite arene rings are twisted to each other at an angle of 74.41 (17)°. Intra­molecular O—H⋯O hydrogen bonds help to stabilize the mol­ecular structure. In the crystal, a C—H⋯π inter­action occurs. One of the *tert*-butyl groups is disordered over two sets of sites with a site-occupancy ratio of 0.70:0.30.

## Related literature

For background to the title compound, see: Csokai *et al.* (2006 ▶); Casnati *et al.* (1995 ▶). For the synthesis, see: Li *et al.* (1999 ▶).
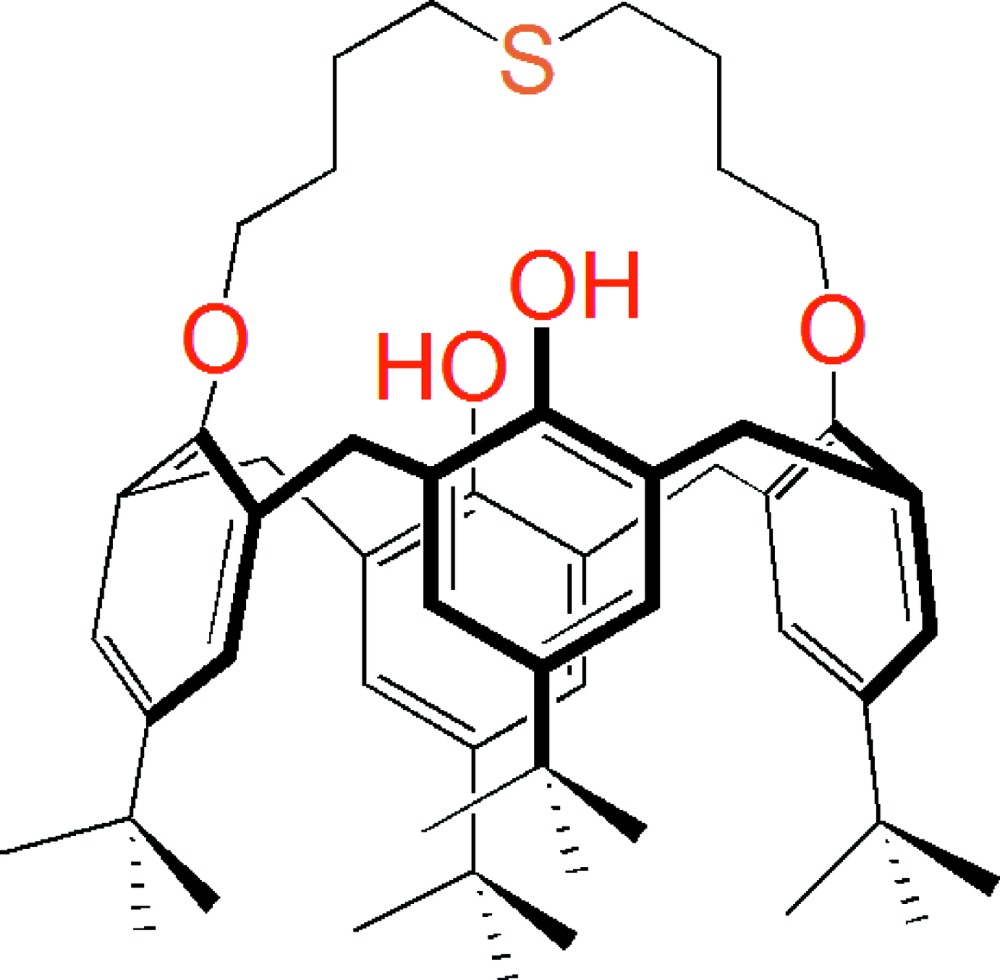


## Experimental

### 

#### Crystal data

C_52_H_70_O_4_S
*M* *_r_* = 791.14
Orthorhombic, 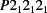

*a* = 10.6222 (8) Å
*b* = 18.4690 (14) Å
*c* = 24.5375 (18) Å
*V* = 4813.8 (6) Å^3^
*Z* = 4
Mo *K*α radiation
μ = 0.11 mm^−1^
*T* = 293 K
0.40 × 0.32 × 0.31 mm

#### Data collection

Bruker SMART 1000 CCD area-detector diffractometer
25550 measured reflections
8942 independent reflections
5384 reflections with *I* > 2σ(*I*)
*R*_int_ = 0.048

#### Refinement

*R*[*F* ^2^ > 2σ(*F* ^2^)] = 0.066
*wR*(*F* ^2^) = 0.191
*S* = 0.93
8942 reflections
562 parameters
78 restraints
H atoms treated by a mixture of independent and constrained refinement
Δρ_max_ = 0.56 e Å^−3^
Δρ_min_ = −0.33 e Å^−3^
Absolute structure: Flack (1983 ▶), 3232 Friedel pairs
Flack parameter: −0.09 (18)

### 

Data collection: *SMART* (Bruker, 2007 ▶); cell refinement: *SAINT* (Bruker, 2007 ▶); data reduction: *SAINT*; program(s) used to solve structure: *SHELXTL* (Sheldrick, 2008 ▶); program(s) used to refine structure: *SHELXTL*; molecular graphics: *SHELXTL*; software used to prepare material for publication: *SHELXTL*.

## Supplementary Material

Crystal structure: contains datablock(s) I, global. DOI: 10.1107/S1600536813008684/xu5679sup1.cif

Structure factors: contains datablock(s) I. DOI: 10.1107/S1600536813008684/xu5679Isup2.hkl

Supplementary material file. DOI: 10.1107/S1600536813008684/xu5679Isup3.cdx

Supplementary material file. DOI: 10.1107/S1600536813008684/xu5679Isup4.cml

Additional supplementary materials:  crystallographic information; 3D view; checkCIF report

## Figures and Tables

**Table 1:** Hydrogen-bond geometry (Å, °) *Cg* is the centroid of the C30–C35 benzene ring.

*D*—H⋯*A*	*D*—H	H⋯*A*	*D*⋯*A*	*D*—H⋯*A*
O3—H3⋯O1	0.80 (2)	2.02 (3)	2.764 (4)	154 (5)
O4—H4⋯O2	0.82 (2)	2.16 (4)	2.897 (4)	150 (7)
C6—H6*B*⋯*Cg* ^i^	0.97	2.88	3.806 (6)	159

Symmetry code: (i) 
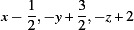
.

**Fig. 1.**
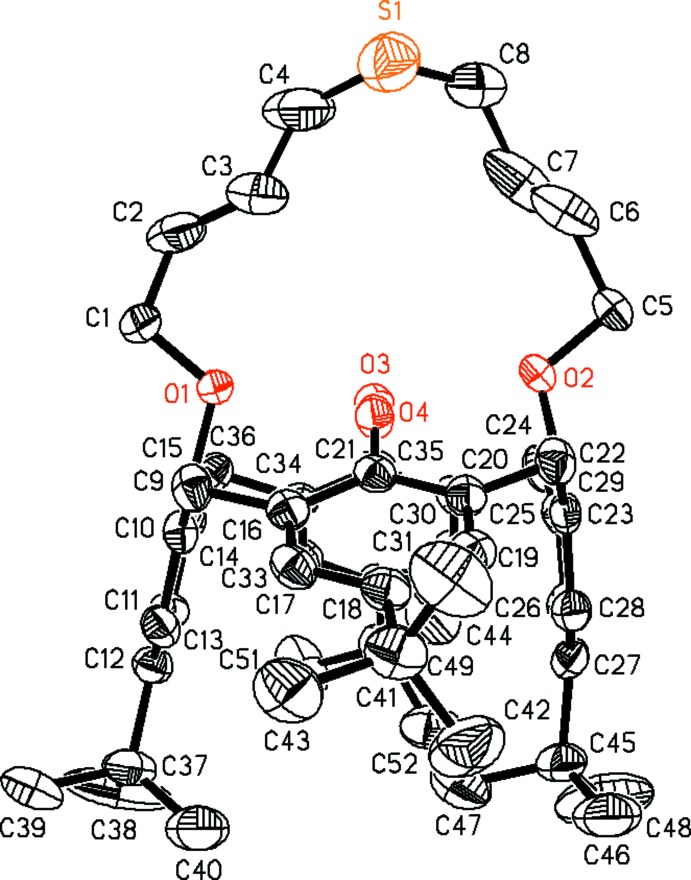
The structure of the title compound.

## supplementary crystallographic information

### Comment

In particular, Calix-Crown ethers in which the proper-sized crown rings are incorporated into the calixarene framework have attracted intense interest as a selective extractant for specific metal ions (Casnati *et al.*, 1995; Csokai *et al.*, 2006)). In this work, the title *p*-*tert*-butylcalixarene-thiacrown in the cone conformation was synthesized, and this is the first report of the crystal structure for the calixarene-thiacrown.

The title compound (I) is shown in Fig 1. The calixarene retains a distorted cone conformation in the solid state. The opposite arene rings A and C that bear the substituents are almost parallel to each other, whereas the other rings B and D include dihedral angles of 74.41 (17)°. Moreover, the plane defined by four methylenic bridges was chosen as a reference plane. The two rings bear the substituents are more vertical than the other two rings. The carbon atoms C1 and C5 in the alkyl chain points towards the exterior of the macrocycle and the torsion angles around the O1—C1, O2—C5 and bonds do not deviate from ideal *syn* values by about 5°. An additional close intramolecular hydrogen bonds between O4 and O2, O3 and O1 stabilize this conformation.

### Experimental

A mixture of *p-tert-*butylcalix[4]arene dibromides (Li *et al.*, 1999) (137 mg, 0.15 mmol) and Na_2_S.9H_2_O (48 mg, 0.20 mmol) in DMF (5 ml) was stirred at 333 K for 5 h. After cooling the reaction mixture to room temperature, it was quenched by water and extracted with dichloromethane. The organic layer was then washed with brine, dried over Na_2_SO_4_, and filtered. The solvent was evaporated *in vacuo*, and the residue was purified by column chromatography on silica gel from petroleum ether/dichloromethane. Petroleum ether/dichloromethane (5:1, *v*/*v*) to give 98 mg (83%) of compound I as white solid: mp 561~564 K; ^1^H NMR (400 MHz, CDCl_3_): *d* 7.92 (s, 2H), 7.05 (s, 4H), 6.85 (s, 4H), 4.26 (d, *J* = 12.8 Hz, 4H), 4.02 (t, *J* = 5.2 Hz, 4H), 3.32 (d, *J* = 12.8 Hz, 4H), 2.77 (brs, 4H), 2.31 (m, 4H), 2.06 (m, 4H), 1.28 (s, 18H), 1.00 (s, 18H); ^13^C NMR (100 MHz, CDCl_3_): *d*: 150.89 (C), 149.76 (C), 146.84 (C), 141.26 (C), 132.71 (C), 127.42 (C), 125.46 (CH), 125.03 (CH), 75.97 (CH_2_), 53.40 (CH_2_), 33.96 (C), 33.77 (C), 31.81 (CH_2_), 31.69 (CH_3_), 31.01 (CH_3_), 30.09 (CH_2_); MS(MALDI-TOF) *m/z*: 813.5 [*M*+Na]^+^.

Single crystals of (I) suitable for X-ray diffraction analysis were obtained by slow diffusion of petroleum ether into a chloroform solution at 298 K.

### Refinement

Hydroxy H atoms were located in a difference Fourier map and refined isotropically. Other H atoms were positioned geometrically with C—H = 0.93–0.97 Å and allowed to ride on their parent atoms, U_iso_(H) = 1.5U_eq_(C) for methyl H atoms and 1.2U_eq_(C) for the others.

### Crystal data

**Table:**

C_52_H_70_O_4_S	*F*(000) = 1720
*M**_r_* = 791.14	*D*_x_ = 1.092 Mg m^−^^3^
Orthorhombic, *P*2_1_2_1_2_1_	Mo *K*α radiation, λ = 0.71073 Å
Hall symbol: P 2ac 2ab	Cell parameters from 4874 reflections
*a* = 10.6222 (8) Å	θ = 2.2–19.9°
*b* = 18.4690 (14) Å	µ = 0.11 mm^−^^1^
*c* = 24.5375 (18) Å	*T* = 293 K
*V* = 4813.8 (6) Å^3^	Prismatic, colorless
*Z* = 4	0.40 × 0.32 × 0.31 mm

### Data collection

**Table:**

Bruker SMART 1000 CCD area-detector diffractometer	5384 reflections with *I* > 2σ(*I*)
Radiation source: fine-focus sealed tube	*R*_int_ = 0.048
Graphite monochromator	θ_max_ = 25.5°, θ_min_ = 2.0°
phi and ω scans	*h* = −12→12
25550 measured reflections	*k* = −22→22
8942 independent reflections	*l* = −29→23

### Refinement

**Table:**

Refinement on *F*^2^	Secondary atom site location: difference Fourier map
Least-squares matrix: full	Hydrogen site location: inferred from neighbouring sites
*R*[*F*^2^ > 2σ(*F*^2^)] = 0.066	H atoms treated by a mixture of independent and constrained refinement
*wR*(*F*^2^) = 0.191	*w* = 1/[σ^2^(*F*_o_^2^) + (0.1159*P*)^2^] where *P* = (*F*_o_^2^ + 2*F*_c_^2^)/3
*S* = 0.93	(Δ/σ)_max_ = 0.002
8942 reflections	Δρ_max_ = 0.56 e Å^−^^3^
562 parameters	Δρ_min_ = −0.33 e Å^−^^3^
78 restraints	Absolute structure: Flack (1983), 3232 Friedel pairs
Primary atom site location: structure-invariant direct methods	Flack parameter: −0.09 (18)

### Special details

**Table:**

Geometry. All e.s.d.'s (except the e.s.d. in the dihedral angle between two l.s. planes) are estimated using the full covariance matrix. The cell e.s.d.'s are taken into account individually in the estimation of e.s.d.'s in distances, angles and torsion angles; correlations between e.s.d.'s in cell parameters are only used when they are defined by crystal symmetry. An approximate (isotropic) treatment of cell e.s.d.'s is used for estimating e.s.d.'s involving l.s. planes.
Refinement. Refinement of *F*^2^ against ALL reflections. The weighted *R*-factor *wR* and goodness of fit *S* are based on *F*^2^, conventional *R*-factors *R* are based on *F*, with *F* set to zero for negative *F*^2^. The threshold expression of *F*^2^ > σ(*F*^2^) is used only for calculating *R*-factors(gt) *etc*. and is not relevant to the choice of reflections for refinement. *R*-factors based on *F*^2^ are statistically about twice as large as those based on *F*, and *R*- factors based on ALL data will be even larger.

### Fractional atomic coordinates and isotropic or equivalent isotropic displacement parameters (Å^2^)

**Table:**

	*x*	*y*	*z*	*U*_iso_*/*U*_eq_	Occ. (<1)
S1	0.0878 (2)	0.94377 (10)	0.90988 (9)	0.1381 (7)	
O1	0.2336 (2)	0.69951 (12)	0.81364 (10)	0.0593 (6)	
O2	−0.1025 (2)	0.69865 (13)	0.93842 (10)	0.0637 (7)	
O3	−0.0257 (3)	0.69895 (14)	0.82319 (10)	0.0590 (6)	
O4	0.1556 (3)	0.65281 (13)	0.92220 (11)	0.0617 (7)	
C1	0.3305 (4)	0.74876 (19)	0.7971 (2)	0.0758 (12)	
H1A	0.3130	0.7667	0.7607	0.091*	
H1B	0.4110	0.7240	0.7964	0.091*	
C2	0.3354 (7)	0.8098 (3)	0.8359 (4)	0.159 (2)	
H2A	0.3804	0.7928	0.8678	0.191*	
H2B	0.3874	0.8470	0.8193	0.191*	
C3	0.2230 (7)	0.8448 (3)	0.8550 (4)	0.157 (2)	
H3A	0.1679	0.8069	0.8684	0.188*	
H3B	0.1821	0.8655	0.8233	0.188*	
C4	0.2248 (8)	0.9005 (3)	0.8962 (4)	0.164 (2)	
H4A	0.2862	0.9366	0.8853	0.196*	
H4B	0.2548	0.8789	0.9298	0.196*	
C5	−0.1742 (5)	0.7463 (2)	0.9726 (2)	0.0911 (14)	
H5A	−0.1618	0.7346	1.0107	0.109*	
H5B	−0.2632	0.7431	0.9642	0.109*	
C6	−0.1213 (6)	0.8256 (3)	0.9591 (3)	0.1138 (15)	
H6A	−0.1137	0.8319	0.9200	0.137*	
H6B	−0.1787	0.8621	0.9730	0.137*	
C7	−0.0010 (6)	0.8333 (3)	0.9843 (3)	0.1219 (16)	
H7A	0.0567	0.7974	0.9699	0.146*	
H7B	−0.0085	0.8257	1.0233	0.146*	
C8	0.0510 (7)	0.9105 (3)	0.9730 (3)	0.1232 (18)	
H8A	0.1268	0.9152	0.9948	0.148*	
H8B	−0.0098	0.9439	0.9884	0.148*	
C9	0.2391 (3)	0.62980 (17)	0.79171 (13)	0.0477 (8)	
C10	0.3048 (3)	0.57645 (17)	0.81889 (13)	0.0477 (8)	
C11	0.3061 (3)	0.50807 (18)	0.79627 (14)	0.0545 (9)	
H11	0.3521	0.4720	0.8137	0.065*	
C12	0.2432 (4)	0.49106 (18)	0.74970 (15)	0.0574 (9)	
C13	0.1713 (4)	0.5451 (2)	0.72468 (14)	0.0581 (9)	
H13	0.1246	0.5337	0.6938	0.070*	
C14	0.1680 (3)	0.61492 (19)	0.74485 (13)	0.0515 (8)	
C15	0.0867 (4)	0.6714 (2)	0.71783 (14)	0.0605 (10)	
H15A	0.1150	0.7188	0.7296	0.073*	
H15B	0.0997	0.6686	0.6788	0.073*	
C16	−0.0521 (4)	0.66559 (17)	0.72893 (14)	0.0534 (9)	
C17	−0.1379 (4)	0.64985 (19)	0.68798 (15)	0.0604 (10)	
H17	−0.1071	0.6419	0.6530	0.072*	
C18	−0.2676 (4)	0.64519 (18)	0.69618 (16)	0.0607 (10)	
C19	−0.3085 (4)	0.65502 (18)	0.74975 (16)	0.0625 (10)	
H19	−0.3939	0.6504	0.7572	0.075*	
C20	−0.2279 (3)	0.67124 (17)	0.79206 (14)	0.0517 (8)	
C21	−0.1006 (4)	0.67838 (17)	0.78106 (13)	0.0503 (8)	
C22	−0.2760 (4)	0.6799 (2)	0.84908 (15)	0.0632 (10)	
H22A	−0.3673	0.6807	0.8486	0.076*	
H22B	−0.2471	0.7258	0.8637	0.076*	
C23	−0.2312 (3)	0.61877 (19)	0.88562 (14)	0.0537 (9)	
C24	−0.1513 (3)	0.63011 (19)	0.92911 (13)	0.0528 (9)	
C25	−0.1022 (4)	0.5723 (2)	0.95819 (14)	0.0597 (9)	
C26	−0.1364 (4)	0.5030 (2)	0.94321 (16)	0.0646 (10)	
H26	−0.1059	0.4643	0.9635	0.078*	
C27	−0.2141 (4)	0.4887 (2)	0.89929 (17)	0.0649 (10)	
C28	−0.2613 (4)	0.54823 (19)	0.87183 (15)	0.0580 (9)	
H28	−0.3159	0.5405	0.8428	0.070*	
C29	−0.0038 (4)	0.5823 (2)	1.00247 (15)	0.0737 (11)	
H29A	−0.0300	0.5573	1.0354	0.088*	
H29B	0.0057	0.6333	1.0109	0.088*	
C30	0.1205 (4)	0.5515 (2)	0.98240 (14)	0.0611 (10)	
C31	0.1629 (4)	0.4873 (2)	1.00226 (15)	0.0696 (11)	
H31	0.1182	0.4656	1.0305	0.084*	
C32	0.2693 (4)	0.4524 (2)	0.98259 (15)	0.0681 (10)	
C33	0.3335 (4)	0.4875 (2)	0.94081 (15)	0.0625 (10)	
H33	0.4047	0.4655	0.9262	0.075*	
C34	0.2958 (3)	0.55424 (18)	0.91986 (13)	0.0514 (8)	
C35	0.1904 (4)	0.58580 (18)	0.94134 (13)	0.0531 (8)	
C36	0.3663 (3)	0.58945 (19)	0.87391 (14)	0.0553 (9)	
H36A	0.4516	0.5707	0.8731	0.066*	
H36B	0.3711	0.6412	0.8805	0.066*	
C37	0.2451 (5)	0.4141 (2)	0.7254 (2)	0.0834 (13)	
C38	0.3348 (10)	0.3659 (3)	0.7538 (4)	0.211 (5)	
H38A	0.3105	0.3611	0.7913	0.317*	
H38B	0.4179	0.3861	0.7517	0.317*	
H38C	0.3343	0.3191	0.7367	0.317*	
C39	0.2931 (8)	0.4212 (3)	0.6637 (3)	0.148 (3)	
H39A	0.3701	0.4483	0.6629	0.222*	
H39B	0.2304	0.4456	0.6424	0.222*	
H39C	0.3074	0.3737	0.6489	0.222*	
C40	0.1169 (6)	0.3839 (3)	0.7209 (3)	0.129 (2)	
H40A	0.1211	0.3363	0.7053	0.194*	
H40B	0.0667	0.4146	0.6979	0.194*	
H40C	0.0794	0.3812	0.7564	0.194*	
C41	−0.3635 (5)	0.6318 (3)	0.65089 (19)	0.0860 (14)	
C42	−0.4427 (6)	0.5666 (4)	0.6640 (3)	0.139 (2)	
H42A	−0.3904	0.5242	0.6646	0.208*	
H42B	−0.5068	0.5610	0.6368	0.208*	
H42C	−0.4813	0.5729	0.6991	0.208*	
C43	−0.3031 (6)	0.6218 (3)	0.5947 (2)	0.1106 (18)	
H43A	−0.2541	0.5782	0.5945	0.166*	
H43B	−0.2495	0.6624	0.5869	0.166*	
H43C	−0.3678	0.6187	0.5675	0.166*	
C44	−0.4535 (6)	0.6987 (4)	0.6479 (3)	0.134 (2)	
H44A	−0.5107	0.6928	0.6179	0.201*	
H44B	−0.4047	0.7419	0.6426	0.201*	
H44C	−0.5002	0.7025	0.6812	0.201*	
C45	−0.2315 (5)	0.4123 (2)	0.8778 (2)	0.0913 (15)	
C46	−0.2322 (12)	0.3563 (4)	0.9232 (4)	0.220 (5)	
H46A	−0.2786	0.3145	0.9116	0.330*	
H46B	−0.2713	0.3766	0.9550	0.330*	
H46C	−0.1473	0.3426	0.9317	0.330*	
C47	−0.1257 (9)	0.3966 (4)	0.8396 (5)	0.242 (7)	
H47A	−0.1399	0.3508	0.8221	0.362*	
H47B	−0.0480	0.3950	0.8595	0.362*	
H47C	−0.1213	0.4341	0.8125	0.362*	
C48	−0.3565 (7)	0.4035 (3)	0.8463 (3)	0.136 (2)	
H48A	−0.3520	0.4300	0.8127	0.203*	
H48B	−0.4246	0.4219	0.8680	0.203*	
H48C	−0.3706	0.3532	0.8387	0.203*	
C49	0.3076 (5)	0.3784 (2)	1.00480 (19)	0.0905 (14)	
C50	0.3172 (12)	0.3800 (5)	1.0656 (3)	0.125 (3)	0.70
H50A	0.3239	0.3314	1.0792	0.188*	0.70
H50B	0.2435	0.4026	1.0806	0.188*	0.70
H50C	0.3905	0.4070	1.0761	0.188*	0.70
C51	0.4261 (11)	0.3498 (6)	0.9809 (4)	0.137 (3)	0.70
H51A	0.4936	0.3830	0.9880	0.206*	0.70
H51B	0.4159	0.3443	0.9422	0.206*	0.70
H51C	0.4452	0.3037	0.9969	0.206*	0.70
C52	0.1961 (10)	0.3227 (4)	0.9911 (4)	0.132 (3)	0.70
H52A	0.2204	0.2749	1.0021	0.198*	0.70
H52B	0.1799	0.3232	0.9526	0.198*	0.70
H52C	0.1214	0.3369	1.0104	0.198*	0.70
C50'	0.235 (2)	0.3516 (12)	1.0525 (9)	0.132 (4)	0.30
H50D	0.1756	0.3886	1.0621	0.199*	0.30
H50E	0.2871	0.3409	1.0834	0.199*	0.30
H50F	0.1907	0.3087	1.0416	0.199*	0.30
C51'	0.4478 (18)	0.3901 (11)	1.0275 (10)	0.132 (4)	0.30
H51D	0.4391	0.3964	1.0661	0.198*	0.30
H51E	0.4862	0.4325	1.0120	0.198*	0.30
H51F	0.4998	0.3487	1.0203	0.198*	0.30
C52'	0.333 (3)	0.3288 (9)	0.9581 (8)	0.124 (4)	0.30
H52D	0.3845	0.2894	0.9701	0.185*	0.30
H52E	0.3749	0.3549	0.9297	0.185*	0.30
H52F	0.2542	0.3102	0.9445	0.185*	0.30
H3	0.046 (2)	0.713 (2)	0.8223 (18)	0.086 (16)*	
H4	0.096 (5)	0.674 (3)	0.936 (3)	0.17 (3)*	

### Atomic displacement parameters (Å^2^)

**Table:**

	*U*^11^	*U*^22^	*U*^33^	*U*^12^	*U*^13^	*U*^23^
S1	0.1379 (15)	0.1169 (13)	0.1593 (16)	0.0001 (12)	0.0094 (13)	0.0160 (11)
O1	0.0668 (16)	0.0406 (12)	0.0704 (15)	−0.0052 (11)	0.0217 (13)	−0.0028 (11)
O2	0.0686 (16)	0.0533 (14)	0.0691 (16)	0.0041 (13)	0.0218 (13)	−0.0159 (12)
O3	0.0531 (17)	0.0683 (16)	0.0556 (15)	0.0007 (14)	0.0047 (13)	−0.0041 (12)
O4	0.0675 (18)	0.0528 (15)	0.0648 (16)	0.0109 (13)	0.0056 (13)	0.0052 (12)
C1	0.078 (3)	0.052 (2)	0.098 (3)	−0.007 (2)	0.027 (3)	0.000 (2)
C2	0.148 (5)	0.078 (3)	0.250 (6)	−0.042 (3)	0.060 (5)	−0.045 (4)
C3	0.159 (4)	0.073 (3)	0.238 (5)	−0.024 (3)	0.053 (4)	−0.046 (3)
C4	0.170 (5)	0.080 (3)	0.241 (6)	−0.027 (4)	0.038 (5)	−0.042 (4)
C5	0.091 (3)	0.080 (3)	0.102 (3)	0.003 (2)	0.033 (3)	−0.037 (2)
C6	0.119 (3)	0.096 (3)	0.126 (3)	0.020 (3)	0.006 (3)	−0.045 (3)
C7	0.131 (4)	0.107 (3)	0.128 (3)	0.022 (3)	−0.005 (3)	−0.042 (3)
C8	0.139 (4)	0.097 (3)	0.133 (4)	0.003 (3)	0.004 (3)	−0.023 (3)
C9	0.051 (2)	0.0442 (18)	0.0479 (19)	−0.0015 (15)	0.0128 (16)	−0.0030 (14)
C10	0.0425 (18)	0.0474 (18)	0.0532 (19)	−0.0031 (15)	0.0033 (15)	−0.0011 (15)
C11	0.055 (2)	0.0477 (19)	0.061 (2)	0.0068 (16)	−0.0048 (17)	−0.0014 (16)
C12	0.057 (2)	0.0495 (19)	0.066 (2)	0.0027 (17)	−0.0008 (19)	−0.0092 (17)
C13	0.059 (2)	0.064 (2)	0.051 (2)	0.0037 (19)	0.0044 (17)	−0.0102 (17)
C14	0.049 (2)	0.061 (2)	0.0453 (19)	0.0044 (16)	0.0064 (16)	0.0024 (16)
C15	0.067 (3)	0.063 (2)	0.051 (2)	0.0097 (19)	0.0092 (18)	0.0158 (17)
C16	0.066 (2)	0.0399 (18)	0.054 (2)	0.0108 (16)	0.0034 (18)	0.0111 (15)
C17	0.077 (3)	0.056 (2)	0.048 (2)	0.0158 (18)	0.002 (2)	−0.0001 (16)
C18	0.066 (3)	0.051 (2)	0.066 (2)	0.0094 (17)	−0.008 (2)	−0.0015 (17)
C19	0.059 (2)	0.054 (2)	0.075 (3)	0.0078 (17)	−0.004 (2)	0.0038 (19)
C20	0.055 (2)	0.0440 (18)	0.056 (2)	0.0085 (16)	0.0009 (18)	0.0057 (15)
C21	0.060 (2)	0.0407 (18)	0.050 (2)	0.0088 (16)	0.0002 (18)	0.0035 (14)
C22	0.053 (2)	0.064 (2)	0.072 (2)	0.0151 (18)	0.0076 (19)	0.0092 (19)
C23	0.048 (2)	0.057 (2)	0.056 (2)	0.0056 (17)	0.0177 (17)	0.0003 (16)
C24	0.056 (2)	0.057 (2)	0.046 (2)	0.0024 (17)	0.0118 (17)	−0.0019 (16)
C25	0.059 (2)	0.067 (2)	0.053 (2)	0.0043 (19)	0.0160 (18)	−0.0014 (18)
C26	0.070 (3)	0.064 (2)	0.060 (2)	0.0077 (19)	0.014 (2)	0.0148 (19)
C27	0.059 (2)	0.052 (2)	0.084 (3)	−0.0064 (18)	0.017 (2)	0.0058 (19)
C28	0.052 (2)	0.061 (2)	0.061 (2)	0.0008 (18)	−0.0003 (17)	0.0006 (17)
C29	0.074 (3)	0.094 (3)	0.053 (2)	0.013 (2)	0.006 (2)	−0.005 (2)
C30	0.071 (3)	0.068 (2)	0.0442 (19)	0.010 (2)	−0.0014 (18)	−0.0040 (17)
C31	0.085 (3)	0.077 (3)	0.047 (2)	0.009 (2)	0.008 (2)	0.0075 (19)
C32	0.089 (3)	0.062 (2)	0.054 (2)	0.008 (2)	−0.002 (2)	0.0046 (18)
C33	0.069 (2)	0.065 (2)	0.053 (2)	0.0162 (19)	0.0001 (19)	−0.0013 (18)
C34	0.056 (2)	0.0507 (19)	0.0477 (19)	−0.0047 (17)	−0.0074 (16)	−0.0087 (15)
C35	0.062 (2)	0.0522 (19)	0.0448 (19)	0.0035 (17)	−0.0081 (17)	−0.0055 (16)
C36	0.053 (2)	0.057 (2)	0.056 (2)	−0.0015 (17)	−0.0019 (17)	0.0028 (16)
C37	0.085 (3)	0.058 (2)	0.107 (4)	0.001 (2)	−0.012 (3)	−0.029 (2)
C38	0.284 (11)	0.081 (4)	0.269 (10)	0.087 (6)	−0.171 (9)	−0.087 (5)
C39	0.200 (8)	0.101 (4)	0.143 (5)	−0.021 (5)	0.042 (5)	−0.072 (4)
C40	0.120 (5)	0.087 (4)	0.180 (6)	−0.015 (4)	0.001 (5)	−0.038 (4)
C41	0.088 (3)	0.091 (3)	0.079 (3)	0.005 (3)	−0.025 (3)	−0.007 (2)
C42	0.140 (6)	0.148 (6)	0.130 (5)	−0.058 (5)	−0.020 (4)	−0.024 (4)
C43	0.126 (5)	0.133 (5)	0.073 (3)	−0.008 (4)	−0.020 (3)	−0.016 (3)
C44	0.124 (5)	0.157 (6)	0.121 (4)	0.049 (4)	−0.061 (4)	−0.028 (4)
C45	0.095 (4)	0.051 (2)	0.128 (4)	−0.009 (2)	0.013 (3)	−0.009 (2)
C46	0.324 (14)	0.076 (4)	0.261 (10)	−0.048 (6)	−0.080 (10)	0.046 (5)
C47	0.176 (9)	0.143 (7)	0.406 (16)	−0.032 (6)	0.128 (10)	−0.149 (9)
C48	0.149 (6)	0.076 (4)	0.182 (6)	−0.018 (4)	−0.009 (5)	−0.037 (4)
C49	0.114 (4)	0.072 (3)	0.085 (3)	0.024 (3)	0.005 (3)	0.016 (2)
C50	0.173 (8)	0.102 (6)	0.100 (5)	0.024 (5)	−0.040 (5)	0.036 (4)
C51	0.177 (8)	0.105 (6)	0.131 (7)	0.066 (6)	0.002 (6)	0.028 (5)
C52	0.183 (8)	0.072 (4)	0.141 (6)	0.011 (5)	−0.031 (6)	0.018 (4)
C50'	0.180 (10)	0.093 (8)	0.124 (8)	0.013 (8)	−0.016 (8)	0.036 (7)
C51'	0.172 (9)	0.096 (7)	0.129 (8)	0.048 (7)	−0.026 (8)	0.025 (7)
C52'	0.178 (10)	0.065 (6)	0.128 (8)	0.033 (8)	−0.017 (8)	−0.004 (7)

### Geometric parameters (Å, º)

**Table:**

S1—C4	1.694 (8)	C30—C35	1.403 (5)
S1—C8	1.711 (7)	C31—C32	1.387 (6)
O1—C9	1.397 (4)	C31—H31	0.9300
O1—C1	1.433 (5)	C32—C33	1.391 (5)
O2—C24	1.387 (4)	C32—C49	1.527 (6)
O2—C5	1.435 (4)	C33—C34	1.395 (5)
O3—C21	1.359 (4)	C33—H33	0.9300
O3—H3	0.80 (2)	C34—C35	1.367 (5)
O4—C35	1.374 (4)	C34—C36	1.502 (5)
O4—H4	0.82 (2)	C36—H36A	0.9700
C1—C2	1.476 (7)	C36—H36B	0.9700
C1—H1A	0.9700	C37—C40	1.476 (7)
C1—H1B	0.9700	C37—C38	1.479 (8)
C2—C3	1.437 (8)	C37—C39	1.603 (8)
C2—H2A	0.9700	C38—H38A	0.9600
C2—H2B	0.9700	C38—H38B	0.9600
C3—C4	1.441 (9)	C38—H38C	0.9600
C3—H3A	0.9700	C39—H39A	0.9600
C3—H3B	0.9700	C39—H39B	0.9600
C4—H4A	0.9700	C39—H39C	0.9600
C4—H4B	0.9700	C40—H40A	0.9600
C5—C6	1.602 (7)	C40—H40B	0.9600
C5—H5A	0.9700	C40—H40C	0.9600
C5—H5B	0.9700	C41—C42	1.504 (7)
C6—C7	1.427 (8)	C41—C43	1.532 (7)
C6—H6A	0.9700	C41—C44	1.563 (7)
C6—H6B	0.9700	C42—H42A	0.9600
C7—C8	1.554 (8)	C42—H42B	0.9600
C7—H7A	0.9700	C42—H42C	0.9600
C7—H7B	0.9700	C43—H43A	0.9600
C8—H8A	0.9700	C43—H43B	0.9600
C8—H8B	0.9700	C43—H43C	0.9600
C9—C10	1.379 (5)	C44—H44A	0.9600
C9—C14	1.403 (5)	C44—H44B	0.9600
C10—C11	1.380 (4)	C44—H44C	0.9600
C10—C36	1.519 (5)	C45—C47	1.492 (10)
C11—C12	1.361 (5)	C45—C46	1.519 (9)
C11—H11	0.9300	C45—C48	1.546 (8)
C12—C13	1.398 (5)	C46—H46A	0.9600
C12—C37	1.541 (5)	C46—H46B	0.9600
C13—C14	1.382 (5)	C46—H46C	0.9600
C13—H13	0.9300	C47—H47A	0.9600
C14—C15	1.508 (5)	C47—H47B	0.9600
C15—C16	1.503 (5)	C47—H47C	0.9600
C15—H15A	0.9700	C48—H48A	0.9600
C15—H15B	0.9700	C48—H48B	0.9600
C16—C17	1.387 (5)	C48—H48C	0.9600
C16—C21	1.399 (5)	C49—C50'	1.486 (16)
C17—C18	1.396 (5)	C49—C51	1.486 (10)
C17—H17	0.9300	C49—C52'	1.491 (15)
C18—C19	1.396 (5)	C49—C50	1.496 (9)
C18—C41	1.528 (6)	C49—C52	1.604 (10)
C19—C20	1.378 (5)	C49—C51'	1.605 (16)
C19—H19	0.9300	C50—H50A	0.9600
C20—C21	1.386 (5)	C50—H50B	0.9600
C20—C22	1.498 (5)	C50—H50C	0.9600
C22—C23	1.519 (5)	C50—H50E	0.9032
C22—H22A	0.9700	C51—H51A	0.9600
C22—H22B	0.9700	C51—H51B	0.9600
C23—C24	1.379 (5)	C51—H51C	0.9600
C23—C28	1.384 (5)	C52—H52A	0.9600
C24—C25	1.386 (5)	C52—H52B	0.9600
C25—C26	1.380 (5)	C52—H52C	0.9600
C25—C29	1.518 (6)	C50'—H50D	0.9600
C26—C27	1.383 (6)	C50'—H50E	0.9600
C26—H26	0.9300	C50'—H50F	0.9600
C27—C28	1.383 (5)	C51'—H51D	0.9600
C27—C45	1.518 (6)	C51'—H51E	0.9600
C28—H28	0.9300	C51'—H51F	0.9600
C29—C30	1.520 (5)	C52'—H52D	0.9600
C29—H29A	0.9700	C52'—H52E	0.9600
C29—H29B	0.9700	C52'—H52F	0.9600
C30—C31	1.359 (5)		
			
C4—S1—C8	101.9 (4)	C34—C36—H36B	109.1
C9—O1—C1	116.5 (3)	C10—C36—H36B	109.1
C24—O2—C5	117.3 (3)	H36A—C36—H36B	107.8
C21—O3—H3	129 (3)	C40—C37—C38	113.7 (6)
C35—O4—H4	120 (5)	C40—C37—C12	111.5 (4)
O1—C1—C2	109.1 (4)	C38—C37—C12	112.5 (4)
O1—C1—H1A	109.9	C40—C37—C39	104.6 (5)
C2—C1—H1A	109.9	C38—C37—C39	106.7 (6)
O1—C1—H1B	109.9	C12—C37—C39	107.1 (4)
C2—C1—H1B	109.9	C37—C38—H38A	109.5
H1A—C1—H1B	108.3	C37—C38—H38B	109.5
C3—C2—C1	121.7 (6)	H38A—C38—H38B	109.5
C3—C2—H2A	106.9	C37—C38—H38C	109.5
C1—C2—H2A	106.9	H38A—C38—H38C	109.5
C3—C2—H2B	106.9	H38B—C38—H38C	109.5
C1—C2—H2B	106.9	C37—C39—H39A	109.5
H2A—C2—H2B	106.7	C37—C39—H39B	109.5
C2—C3—C4	122.6 (7)	H39A—C39—H39B	109.5
C2—C3—H3A	106.7	C37—C39—H39C	109.5
C4—C3—H3A	106.7	H39A—C39—H39C	109.5
C2—C3—H3B	106.7	H39B—C39—H39C	109.5
C4—C3—H3B	106.7	C37—C40—H40A	109.5
H3A—C3—H3B	106.6	C37—C40—H40B	109.5
C3—C4—S1	117.7 (6)	H40A—C40—H40B	109.5
C3—C4—H4A	107.9	C37—C40—H40C	109.5
S1—C4—H4A	107.9	H40A—C40—H40C	109.5
C3—C4—H4B	107.9	H40B—C40—H40C	109.5
S1—C4—H4B	107.9	C42—C41—C18	110.2 (4)
H4A—C4—H4B	107.2	C42—C41—C43	109.3 (5)
O2—C5—C6	104.8 (4)	C18—C41—C43	113.2 (4)
O2—C5—H5A	110.8	C42—C41—C44	107.5 (5)
C6—C5—H5A	110.8	C18—C41—C44	108.3 (4)
O2—C5—H5B	110.8	C43—C41—C44	108.0 (4)
C6—C5—H5B	110.8	C41—C42—H42A	109.5
H5A—C5—H5B	108.9	C41—C42—H42B	109.5
C7—C6—C5	108.4 (5)	H42A—C42—H42B	109.5
C7—C6—H6A	110.0	C41—C42—H42C	109.5
C5—C6—H6A	110.0	H42A—C42—H42C	109.5
C7—C6—H6B	110.0	H42B—C42—H42C	109.5
C5—C6—H6B	110.0	C41—C43—H43A	109.5
H6A—C6—H6B	108.4	C41—C43—H43B	109.5
C6—C7—C8	109.4 (5)	H43A—C43—H43B	109.5
C6—C7—H7A	109.8	C41—C43—H43C	109.5
C8—C7—H7A	109.8	H43A—C43—H43C	109.5
C6—C7—H7B	109.8	H43B—C43—H43C	109.5
C8—C7—H7B	109.8	C41—C44—H44A	109.5
H7A—C7—H7B	108.2	C41—C44—H44B	109.5
C7—C8—S1	124.9 (4)	H44A—C44—H44B	109.5
C7—C8—H8A	106.1	C41—C44—H44C	109.5
S1—C8—H8A	106.1	H44A—C44—H44C	109.5
C7—C8—H8B	106.1	H44B—C44—H44C	109.5
S1—C8—H8B	106.1	C47—C45—C27	107.9 (4)
H8A—C8—H8B	106.3	C47—C45—C46	109.5 (7)
C10—C9—O1	119.6 (3)	C27—C45—C46	112.3 (5)
C10—C9—C14	121.9 (3)	C47—C45—C48	108.2 (6)
O1—C9—C14	118.3 (3)	C27—C45—C48	112.0 (4)
C9—C10—C11	117.7 (3)	C46—C45—C48	106.9 (6)
C9—C10—C36	122.3 (3)	C45—C46—H46A	109.5
C11—C10—C36	119.8 (3)	C45—C46—H46B	109.5
C12—C11—C10	123.0 (3)	H46A—C46—H46B	109.5
C12—C11—H11	118.5	C45—C46—H46C	109.5
C10—C11—H11	118.5	H46A—C46—H46C	109.5
C11—C12—C13	118.2 (3)	H46B—C46—H46C	109.5
C11—C12—C37	122.1 (3)	C45—C47—H47A	109.5
C13—C12—C37	119.7 (3)	C45—C47—H47B	109.5
C14—C13—C12	121.5 (3)	H47A—C47—H47B	109.5
C14—C13—H13	119.2	C45—C47—H47C	109.5
C12—C13—H13	119.2	H47A—C47—H47C	109.5
C13—C14—C9	117.5 (3)	H47B—C47—H47C	109.5
C13—C14—C15	120.2 (3)	C45—C48—H48A	109.5
C9—C14—C15	122.2 (3)	C45—C48—H48B	109.5
C16—C15—C14	115.6 (3)	H48A—C48—H48B	109.5
C16—C15—H15A	108.4	C45—C48—H48C	109.5
C14—C15—H15A	108.4	H48A—C48—H48C	109.5
C16—C15—H15B	108.4	H48B—C48—H48C	109.5
C14—C15—H15B	108.4	C50'—C49—C51	129.2 (10)
H15A—C15—H15B	107.4	C50'—C49—C52'	119.5 (14)
C17—C16—C21	117.1 (3)	C51—C49—C52'	47.7 (11)
C17—C16—C15	121.9 (3)	C50'—C49—C50	42.0 (10)
C21—C16—C15	121.0 (3)	C51—C49—C50	110.1 (7)
C16—C17—C18	123.9 (3)	C52'—C49—C50	140.0 (9)
C16—C17—H17	118.1	C50'—C49—C32	116.2 (10)
C18—C17—H17	118.1	C51—C49—C32	113.8 (5)
C17—C18—C19	115.8 (4)	C52'—C49—C32	108.9 (8)
C17—C18—C41	124.3 (4)	C50—C49—C32	110.9 (5)
C19—C18—C41	119.9 (4)	C50'—C49—C52	64.5 (11)
C20—C19—C18	123.0 (4)	C51—C49—C52	108.3 (7)
C20—C19—H19	118.5	C52'—C49—C52	64.9 (12)
C18—C19—H19	118.5	C50—C49—C52	105.8 (7)
C19—C20—C21	118.7 (3)	C32—C49—C52	107.6 (4)
C19—C20—C22	121.0 (3)	C50'—C49—C51'	104.7 (14)
C21—C20—C22	120.3 (3)	C51—C49—C51'	53.1 (10)
O3—C21—C20	116.8 (3)	C52'—C49—C51'	100.6 (15)
O3—C21—C16	121.8 (3)	C50—C49—C51'	65.7 (10)
C20—C21—C16	121.4 (3)	C32—C49—C51'	104.5 (8)
C20—C22—C23	111.4 (3)	C52—C49—C51'	147.6 (8)
C20—C22—H22A	109.4	C49—C50—H50A	109.5
C23—C22—H22A	109.4	C49—C50—H50B	109.5
C20—C22—H22B	109.4	C49—C50—H50C	109.5
C23—C22—H22B	109.4	C49—C50—H50D	82.9
H22A—C22—H22B	108.0	H50A—C50—H50D	101.0
C24—C23—C28	118.3 (3)	H50B—C50—H50D	33.5
C24—C23—C22	122.5 (3)	H50C—C50—H50D	140.0
C28—C23—C22	118.9 (3)	C49—C50—H50E	116.3
C23—C24—C25	120.9 (3)	H50A—C50—H50E	27.1
C23—C24—O2	119.7 (3)	H50B—C50—H50E	82.7
C25—C24—O2	118.5 (3)	H50C—C50—H50E	125.0
C26—C25—C24	118.5 (4)	H50D—C50—H50E	76.1
C26—C25—C29	118.9 (4)	C49—C50—H51D	94.6
C24—C25—C29	122.3 (4)	H50A—C50—H51D	97.9
C25—C26—C27	122.8 (4)	H50B—C50—H51D	133.8
C25—C26—H26	118.6	H50C—C50—H51D	24.4
C27—C26—H26	118.6	H50D—C50—H51D	160.6
C28—C27—C26	116.4 (4)	H50E—C50—H51D	121.5
C28—C27—C45	121.7 (4)	C49—C51—H51A	109.5
C26—C27—C45	121.4 (4)	C49—C51—H51B	109.5
C27—C28—C23	123.0 (4)	C49—C51—H51C	109.5
C27—C28—H28	118.5	C49—C51—H51F	103.4
C23—C28—H28	118.5	H51A—C51—H51F	53.0
C25—C29—C30	108.7 (3)	H51B—C51—H51F	146.8
C25—C29—H29A	109.9	H51C—C51—H51F	62.3
C30—C29—H29A	109.9	C49—C52—H52A	109.5
C25—C29—H29B	109.9	C49—C52—H52B	109.5
C30—C29—H29B	109.9	C49—C52—H52C	109.5
H29A—C29—H29B	108.3	C49—C52—H50F	87.7
C31—C30—C35	118.5 (3)	H52A—C52—H50F	63.1
C31—C30—C29	119.8 (4)	H52B—C52—H50F	162.8
C35—C30—C29	121.6 (3)	H52C—C52—H50F	62.4
C30—C31—C32	123.3 (4)	C49—C50'—H50D	107.2
C30—C31—H31	118.3	C49—C50'—H50E	113.1
C32—C31—H31	118.3	H50D—C50'—H50E	109.5
C31—C32—C33	116.1 (3)	C49—C50'—H50F	108.0
C31—C32—C49	120.5 (4)	H50D—C50'—H50F	109.5
C33—C32—C49	123.3 (4)	H50E—C50'—H50F	109.5
C32—C33—C34	122.8 (4)	C49—C51'—H51D	105.6
C32—C33—H33	118.6	C49—C51'—H51E	111.5
C34—C33—H33	118.6	H51D—C51'—H51E	109.5
C35—C34—C33	118.0 (3)	C49—C51'—H51F	111.2
C35—C34—C36	120.9 (3)	H51D—C51'—H51F	109.5
C33—C34—C36	121.1 (3)	H51E—C51'—H51F	109.5
C34—C35—O4	118.2 (3)	C49—C52'—H52D	109.5
C34—C35—C30	121.2 (3)	C49—C52'—H52E	109.5
O4—C35—C30	120.7 (3)	H52D—C52'—H52E	109.5
C34—C36—C10	112.6 (3)	C49—C52'—H52F	109.5
C34—C36—H36A	109.1	H52D—C52'—H52F	109.5
C10—C36—H36A	109.1	H52E—C52'—H52F	109.5
			
C9—O1—C1—C2	−162.1 (5)	C24—C25—C26—C27	2.3 (6)
O1—C1—C2—C3	−44.2 (9)	C29—C25—C26—C27	−172.5 (3)
C1—C2—C3—C4	174.8 (7)	C25—C26—C27—C28	−2.7 (5)
C2—C3—C4—S1	173.2 (7)	C25—C26—C27—C45	169.1 (4)
C8—S1—C4—C3	108.8 (7)	C26—C27—C28—C23	1.9 (5)
C24—O2—C5—C6	−161.3 (4)	C45—C27—C28—C23	−169.9 (4)
O2—C5—C6—C7	−74.7 (5)	C24—C23—C28—C27	−0.7 (5)
C5—C6—C7—C8	−178.7 (4)	C22—C23—C28—C27	173.2 (3)
C6—C7—C8—S1	−63.8 (7)	C26—C25—C29—C30	64.3 (5)
C4—S1—C8—C7	−74.8 (6)	C24—C25—C29—C30	−110.2 (4)
C1—O1—C9—C10	90.5 (4)	C25—C29—C30—C31	−105.5 (4)
C1—O1—C9—C14	−94.9 (4)	C25—C29—C30—C35	70.9 (5)
O1—C9—C10—C11	179.0 (3)	C35—C30—C31—C32	−2.5 (6)
C14—C9—C10—C11	4.6 (5)	C29—C30—C31—C32	174.0 (4)
O1—C9—C10—C36	3.4 (5)	C30—C31—C32—C33	0.7 (6)
C14—C9—C10—C36	−171.0 (3)	C30—C31—C32—C49	−176.8 (4)
C9—C10—C11—C12	−1.8 (5)	C31—C32—C33—C34	0.9 (6)
C36—C10—C11—C12	173.8 (3)	C49—C32—C33—C34	178.3 (4)
C10—C11—C12—C13	−1.8 (6)	C32—C33—C34—C35	−0.5 (5)
C10—C11—C12—C37	−179.5 (4)	C32—C33—C34—C36	−178.7 (3)
C11—C12—C13—C14	2.9 (5)	C33—C34—C35—O4	177.6 (3)
C37—C12—C13—C14	−179.4 (4)	C36—C34—C35—O4	−4.2 (5)
C12—C13—C14—C9	−0.3 (5)	C33—C34—C35—C30	−1.4 (5)
C12—C13—C14—C15	−178.3 (3)	C36—C34—C35—C30	176.8 (3)
C10—C9—C14—C13	−3.5 (5)	C31—C30—C35—C34	2.8 (5)
O1—C9—C14—C13	−178.0 (3)	C29—C30—C35—C34	−173.6 (3)
C10—C9—C14—C15	174.4 (3)	C31—C30—C35—O4	−176.1 (3)
O1—C9—C14—C15	−0.1 (5)	C29—C30—C35—O4	7.4 (5)
C13—C14—C15—C16	75.5 (4)	C35—C34—C36—C10	−79.9 (4)
C9—C14—C15—C16	−102.3 (4)	C33—C34—C36—C10	98.2 (4)
C14—C15—C16—C17	−114.8 (4)	C9—C10—C36—C34	106.0 (4)
C14—C15—C16—C21	67.4 (4)	C11—C10—C36—C34	−69.5 (4)
C21—C16—C17—C18	−0.9 (5)	C11—C12—C37—C40	121.3 (5)
C15—C16—C17—C18	−178.8 (3)	C13—C12—C37—C40	−56.3 (6)
C16—C17—C18—C19	−2.2 (5)	C11—C12—C37—C38	−7.8 (8)
C16—C17—C18—C41	176.6 (4)	C13—C12—C37—C38	174.6 (6)
C17—C18—C19—C20	2.5 (5)	C11—C12—C37—C39	−124.8 (5)
C41—C18—C19—C20	−176.3 (3)	C13—C12—C37—C39	57.6 (6)
C18—C19—C20—C21	0.2 (5)	C17—C18—C41—C42	124.3 (5)
C18—C19—C20—C22	−178.9 (3)	C19—C18—C41—C42	−56.9 (6)
C19—C20—C21—O3	175.9 (3)	C17—C18—C41—C43	1.5 (6)
C22—C20—C21—O3	−5.0 (4)	C19—C18—C41—C43	−179.7 (4)
C19—C20—C21—C16	−3.6 (5)	C17—C18—C41—C44	−118.2 (5)
C22—C20—C21—C16	175.5 (3)	C19—C18—C41—C44	60.5 (6)
C17—C16—C21—O3	−175.5 (3)	C28—C27—C45—C47	87.1 (8)
C15—C16—C21—O3	2.4 (5)	C26—C27—C45—C47	−84.2 (8)
C17—C16—C21—C20	3.9 (5)	C28—C27—C45—C46	−152.2 (6)
C15—C16—C21—C20	−178.2 (3)	C26—C27—C45—C46	36.5 (8)
C19—C20—C22—C23	111.3 (4)	C28—C27—C45—C48	−31.9 (6)
C21—C20—C22—C23	−67.7 (4)	C26—C27—C45—C48	156.8 (5)
C20—C22—C23—C24	115.1 (4)	C31—C32—C49—C50'	−7.6 (14)
C20—C22—C23—C28	−58.4 (4)	C33—C32—C49—C50'	175.1 (13)
C28—C23—C24—C25	0.2 (5)	C31—C32—C49—C51	−177.9 (7)
C22—C23—C24—C25	−173.4 (3)	C33—C32—C49—C51	4.8 (8)
C28—C23—C24—O2	169.2 (3)	C31—C32—C49—C52'	130.9 (13)
C22—C23—C24—O2	−4.4 (5)	C33—C32—C49—C52'	−46.4 (14)
C5—O2—C24—C23	89.4 (4)	C31—C32—C49—C50	−53.2 (8)
C5—O2—C24—C25	−101.3 (4)	C33—C32—C49—C50	129.5 (7)
C23—C24—C25—C26	−1.0 (5)	C31—C32—C49—C52	62.0 (6)
O2—C24—C25—C26	−170.2 (3)	C33—C32—C49—C52	−115.3 (6)
C23—C24—C25—C29	173.6 (3)	C31—C32—C49—C51'	−122.3 (11)
O2—C24—C25—C29	4.4 (5)	C33—C32—C49—C51'	60.4 (11)

### Hydrogen-bond geometry (Å, º)

Cg is the centroid of the C30–C35 benzene ring.

**Table:**

*D*—H···*A*	*D*—H	H···*A*	*D*···*A*	*D*—H···*A*
O3—H3···O1	0.80 (2)	2.02 (3)	2.764 (4)	154 (5)
O4—H4···O2	0.82 (2)	2.16 (4)	2.897 (4)	150 (7)
C6—H6*B*···*Cg*^i^	0.97	2.88	3.806 (6)	159

Symmetry code: (i) *x*−1/2, −*y*+3/2, −*z*+2.
